# Agave Syrup: Chemical Analysis and Nutritional Profile, Applications in the Food Industry and Health Impacts

**DOI:** 10.3390/ijerph19127022

**Published:** 2022-06-08

**Authors:** Ariana Saraiva, Conrado Carrascosa, Fernando Ramos, Dele Raheem, António Raposo

**Affiliations:** 1 Department of Animal Pathology and Production, Bromatology and Food Technology, Faculty of Veterinary, Universidad de Las Palmas de Gran Canaria, Trasmontaña s/n, 35413 Arucas, Spain; ariana_23@outlook.pt (A.S.); conrado.carrascosa@ulpgc.es (C.C.); 2 Pharmacy Faculty, University of Coimbra, Azinhaga de Santa Comba, 3000-548 Coimbra, Portugal; framos@ff.uc.pt; 3 REQUIMTE/LAQV, Rua D. Manuel II, Apartado 55142, 4051-401 Porto, Portugal; 4 Northern Institute for Environmental and Minority Law (NIEM), Arctic Centre, University of Lapland, 96101 Rovaniemi, Finland; braheem@ulapland.fi; 5 CBIOS (Research Center for Biosciences and Health Technologies), Universidade Lusófona de Humanidades e Tecnologias, Campo Grande 376, 1749-024 Lisboa, Portugal

**Keywords:** agave syrup, chemical analysis, food industry, health impacts, nutrition

## Abstract

Agave syrup (AS), a food product made from agave plant sap, is a vegan sweetener that has become popular for replacing conventional sweeteners such as sucrose. As the demand for naturally derived sweeteners has grown in the last decade, this review paper addresses and discusses, in detail, the most relevant aspects of the chemical AS analysis, applications in the food industry, sustainability issues, safety and quality control and, finally, nutritional profile and health impacts. According to our main research outcome, we can assume that the mid-infrared-principal components analysis, high-performance anion exchange chromatography equipped with a pulsed amperometric detector, and thin-layer chromatography can be used to identify and distinguish syrups from natural sources. The main agave–derived products are juice, leaves, bagasse, and fiber. In sustainability terms, it can be stated that certified organic and free trade agave products are the most sustainable options available on the market because they guarantee products being created without pesticides and according to specific labor standards. The Mexican government and AS producers have also established Mexican guidelines which prohibit using any ingredient, sugar or food additive that derives from sources, apart from agave plants, to produce any commercial AS. Due to its nutritional value, AS is a good source of minerals, vitamins and polyphenols compared to other traditional sweeteners. However, further research into the effects of AS on human metabolism is necessary to back its health claims as a natural sugar substitute.

## 1. Introduction

Agave syrup (AS), also referred to as agave nectar, is a recently developed (post-1990) food product made from agave plant sap, particularly *Agave salmiana* and *Agave tequilana*, that is, salmiana and blue agave, respectively. Given its low glycemic index and vegan status, this product has become popular as a substitute for traditional sweeteners such as table sugar (sucrose) and honey [1,2,3].

Fructan hydrolysis produces this natural sweetener. Nectar, which comes as fructans from agave cores (*piñas* in Spanish), is a principal carbohydrate reserve of agave plants. Grown in arid and semi-arid environments, the Agave genus applies photosynthetic adaptation and a crassulacean acid metabolism to periodic water supply. Such plants prevail in Central and Northern America, but a large proportion of the species (about 55%) are located in Mexico, believed to be the centre of agave’s diversity and origin [4]. Both the consumption and socio-economic impacts of agave originate from pre-Columbian times, given its high sugar content [5].

Nowadays, consumers are increasingly becoming familiar with natural ingredients, and the growing prominence of naturalness for consumers has had significant repercussions for the food industry [6,7]. It is feasible that consumers in most countries may reject food products that are not perceived as natural. In recent decades, demand for naturally derived sweeteners has exploded [8].

Based on these premises, this review aims to study the nutritional profile and health impacts of AS consumption, its possible applications in the food industry and sustainability issues, as well as its central safety and quality parameters, including the chemical analysis of its main components.

## 2. Chemical Analysis

Fructose is a sugar present in high contents (approximately 80%) in AS sugars (ASs). The food industry frequently employs it as a sweetener. However, extracting fructose from agave involves fractionation methods, mainly chromatographic techniques, followed by qualitative and quantitative analysis methods [9] NMR (Nuclear Magnetic Resonance), HPLC (High-Performance Liquid Chromatography) and GC-MS (Gas Chromatography Coupled with Mass Spectrometry) are the most widespread analytical methods [9]. Table 1 summarizes the most widespread analytical techniques to characterize ASs, where HPLC is the most followed method. However, these methods are expensive and time-consuming [9]. 

Several authors have developed analytical methods for sugar analysis that are faster, cheaper, and easier than those previously referred to.

Ja et al. (2018) [9] successfully developed a polarimetric method for the fructose-glucose ratio analysis. The obtained results were evaluated and validated by HPLC using fructose and glucose standards. HPLC and the polarimetric method are statistically equivalents in accuracy and reproducibility terms and prove the technical feasibility of the polarimetric method. This method reduces the equipment required for the fructose-glucose ratio analysis and makes fructose-glucose ratio quantification easier and faster [9]. Thermally untreated and treated *Agave salmiana* syrups have been analyzed by HPLC associated with a refractive index detector, and later by Liquid Chromatography Coupled with Electrospray Ionisation Mass Spectrometry (LC-ESI-MS). The chromatogram profile obtained by HPLC shows the presence of glucose, sucrose, fructose and kestose in the two samples. However, sucrose concentration significantly rises in the thermally treated sample. In both the treated and untreated samples analyzed by LC-ESI-MS, the presence of sucrose, kestose and oligomeric fructans is confirmed. However, fructose and glucose are not detected under the tested conditions. Therefore, authors conclude that, compared to GC-MS, this technique reduces sample preparation times and allows for the analysis of tri- and tetra-saccharides [10].

In an attempt to discriminate ASs from other natural sugars, *Agave tequilana*, *Agave salmiana*, honey, corn and cane syrups have been analyzed by methods of the vibrational spectroscopic type, namely MIR (mid-infrared) and NIR (near-infrared) combined with chemometrics (for example, multivariate data analyses) [11]. Oligosaccharide content and monosaccharide ratios have been evaluated by HPAEC-PAD (High-Performance Anion Exchange Chromatography with a Pulsed Amperometric Detector). This technique is used for sugar analysis thanks to its low detection limits, as is Thin Layer Chromatography (TLC). All the samples show high glucose, fructose and sucrose contents. However, the fructose-glucose ratio can be used to discriminate ASs. The AS analysis by HPAEC-PAD and TLC shows specific sugar profiles, mainly composed of fructose and fructo-oligosaccharides (FOS) compared to other tested syrups. *Agave salmiana* has high sucrose content and *Agave tequilana* exhibits a large quantity of fructose. Hence these techniques can confirm the authenticity of ASs. As vibrational methods, NIR is unable to distinguish the assayed syrups. The combination of MIR spectroscopy and a PCA (Principal Components Analysis) shows significant differences between 1185 and 950 cm^−1^ in the sugar region. Given its high fructose content, in the fructose region *Agave tequilana* syrups display a marked absorption, from 1061 to 1063 cm^−1^, and *Agave salmiana* syrups present high sucrose contents with marked absorption from 997 to 1054 cm^−1^. The other tested syrups also show specific characteristic absorption bands in the carbohydrate’s region. Therefore, MIR-PCA, HPAEC-PAD and TLC can be used to identify and discriminate syrups from natural sources. These methods are fast, nondestructive, simple and economic compared to other techniques (for example, HPLC, GC-MS and NMR) [11].

As a tool to prevent adulteration in ASs and to control their authenticity, Portaluri et al., (2021) [12] developed an approach to detect C4 plants called the 13C site-specific natural isotopic fractionation (SNIF)-NMR approach. After obtaining ethanol from the sugar fermentation of several ASs, it is analyzed via the optimization of a method based on an insensitive nucleus enhanced by a polarization transfer (INEPT) pulse sequence for 13C SNIF-NMR to reduce the acquisition time; it produces reproducible and reliable results. Of 11 commercial ASs, only 1 is authentic. The results also show that maize and cane are converted into sugar syrups, masked by the glucose-fructose ratio. This suggests the probability of using ASs for adulteration [12].

**Table 1:** Analytical methods for the characterization of agave syrup sugars.

Plant	Analytical Method	Detector	Standards	Analysis Conditions	Results	References
*Agave salmiana*	HPLC	Refractive-index	Fructose, arabinose, glucose, lactose, maltose, ribose, galactose, mannose, xylose sucrose and chicory inulin	Stationary phase: column ion exchange; mobile phase: HPLC grade water (flow rate of 0.6 mL/min) Column temperature: 75 °C Run time: 20 min Injection volume: 50 μL	Sugars were well separated with good resolution. Sucrose, glucose and fructose were identified and quantified (85.6 ± 2.52%, 4.67 ± 0.22% and 3.99 ± 0.14% (6.36 ± 0.54%, dry matter), respectively).	[13]
			Arabinose, fructose, galactose, glucose, lactose, maltose, mannose, ribose, sucrose, and xylose	Stationary phase: Zorbax column specific for carbohydrates from the Agilent Mobile phase: 75:25 acetonitrile:water at a flow rate of 1.4 mL/min Column temperature: 30 °C Run time: 15 min Injection volume: 20 μL	Identified sugars: xylose, fructose, glucose, sucrose, maltose. Use of plants in the quiotilla maturity state, including the stem up to its neck, whose fructose concentration was even higher than that presented at its base	[14]
			Fructose, glucose, sucrose, fructo-oligosaccharides standards 1-nystose, 1-β-fructofuranosyl and nystose 1-kestose	Stationary phase: Prevail Carbohydrate ES column Mobile phase: acetonitrile:water (70:30) (1.0 mL/min flow rate) Run time: 18 min Injection volume: 20 μL	Fructose, glucose, sucrose and kestose were identified in thermally untreated agave syrups The sucrose concentration increased in the thermally treated agave syrups The quantity of fructose, glucose and kestose in the agave syrup was similar before/after heat treatment (1.2 and 0.7, 15.21 and 16.12 and 10.89 and 12.71 g L^−1^, respectively)	[10]
	LC	ESI-MS		MS analyses were performed in the [M-H]^−1^ negative mode The nebulizing gas was nitrogen and the damping gas was helium. 3.0 kV spray voltage, 90.0 V capillary voltage Temperature was 250 °C, 10 μL/min flow rate Run time: 7 min Injection volume: 20 μL m/z range acquisition spectra: 50–2000	For the thermally untreated/treated syrups, under the employed conditions the masses that corresponded to glucose and fructose were not identified. The kestose, sucrose and oligomeric fructans were confirmed unambiguously in the untreated/treated agave syrups	
*Agave tequilana*	Total reducing sugars (TRS) and direct reducing sugars (DRS)	-	Fructose corn syrup, fructose and glucose standards.	-	4.4 kg of a fresh head of *A. tequilana* were needed to obtain 1 kg of syrup with 70% TRS and a fructose content of 87.92 ± 1.28%	[15]
*Agave tequilana e A. salmiana*	HPAE	PAD	Fructose, glucose, inositol and mannitol. Fructo-oligosaccharide standard	Monosaccharides analysis: stationary phase: a Dionex CarboPac PA1 column in series was used with a CarboPac PA1 guard column. The mobile phase: isocratic of 80 mM NaOH (1.0 mL/min flow rate) Oligosaccharide analysis: stationary phase: a Dionex CarboPac PA100 column in series was used with a CarboPac PA100 guard column. Mobile phase: solvent A = 160 mM NaOH; solvent B = 160 mM NaOH/1.0 M NaOAc. solvent C = 1.0 M NaOH (0.0 mL/min flow rate)	The main identified monosaccharide was fructose (71.86–92.13% concentration range), followed by glucose (4.73–15.06% concentration range) Fructose-glucose ratio 10:1 Two polyols were detected: one was mannitol (concentration in the ASs went from 0.02% to 2.54%). The other was inositol (0.31–0.43% concentration range) Inulobiose was the main identified oligosaccharide	[16]
	CGC	FID		Oligosaccharide analysis: stationary phase: an Agilent J&W DB-5 (30 m × 0.25 mm, 0.25 μm film thickness; 95% dimethyl–5% diphenyl polysiloxane) open tubular fused-silica capillary column Carrier gas: ultrapure hydrogen (flow rate = 1.2 mL/min) Makeup gas: ultrapure nitrogen (flow rate = 30 mL/min) Injection port temp.: 250 °C Detector temp.: 300 °C		
	^1^H-NMR spectroscopy-PCA	-	-	NMR spectra of syrup samples acquired by the Varian/Agilent 600 MHz AR Premium COMPACTTM spectrophotometer. ^1^H-NMR spectra were measured at 300 K and frequency 599.77 MHz with D_2_O as the solvent, plus an internal reference. The residual HOD signal was employed at 4.9 ppm. The used π/2 pulse was 8.7 μs. The relaxation time was 15 s. There were 16 repetitions.	The *A. salmiana* syrup had an identical profile and another signal at 5.4 ppm, which corresponded to sucrose. The *A. tequilana* syrups showed a greater intensity signal emitted by peaks at 4.0 ppm for fructose, and peaks at 3.8 and 3.7 ppm for sucrose. This method allowed agave syrups to be identified and classified, and was able to differentiate for other natural sweeteners	[17]

## 3. Food Industry Applications and Sustainability Issues

Juice, leaves, bagasse, and fibers are the main products that are derived from agave. The agave industry produces other residue types, such as stalks cuticles and spines with relevant cellulose and bioactive compounds. This section contemplates the utilization and sustainability of ASs as a main product in the food industry.

Industrial (nonalcoholic) AS production is similar to that of the tequila procedure (40–50% alcohol or 80–100 US proof). The exceptions are additional fermentation processes and distillation/purification steps. Variability of production methods, type of agave, agave-growing region and the plant part employed in production processes (leaves, pine, sap) produce wide-ranging products sold as ASs.

Since the 17th century, ASs have been used to produce distilled alcohol drinks in Mexico, such as tequila, mezcal, sotol, pulque and henequen, of which Mezcal and tequila are the 2 most popular. The global tequila market is projected to reach $6.36 billion by the end of 2025 [18]. According to the Consejo Regulador del Mezcal, global shipments of mezcal rose by 26% in 2019 [19] (Pattillo, 2021). AS, or nectar, fetched 156 million US$ in 2021 and is projected to fetch 272 million US$ by 2026 [18].

**Figure 1:**
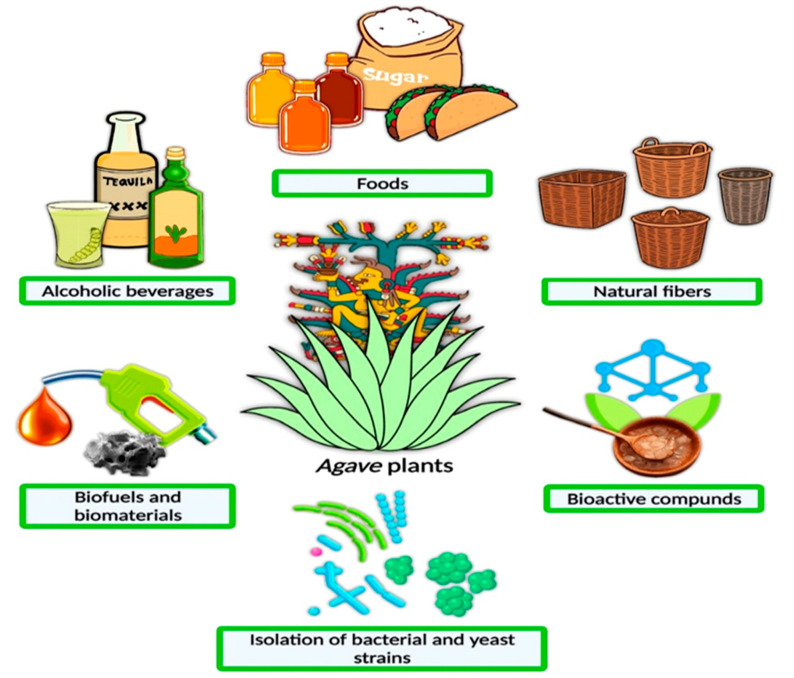
Produce acquired from agave plants [22] (Reprinted with permission from Ref. [22]. Copyright 2021 Elsevier).

The commonest agave species used for AS production are *A. tequilana*, *A. americana*, *A. potatorum*, *A. salmiana* and *A. atrovirens* [20,21]. Several products can be obtained from agave plants (see Figure 1), and these by-products are important sources of income that drive this crop’s cultivation. The agave plant is also utilized in foods such as sugars and syrups, and in Mexican stews [22]. Non-food and non-beverage by-products, such as biofuel and other biomaterials, are presently being questioned in environmental sustainability terms.

The process followed to generate ASs begins by harvesting mature 5–7-year-old blue agave plants. From them, the high carbohydrate contents in plant *piñas* (pine) can be stored. A *piña* looks similar to a pineapple once leaves have been removed. A high-quality piña (weighing up to 68 kg) contains approximately 25–30% *w*/*w* sugars [4]. The next phase consists in milling and crushing piñas for juicy fibers to be obtained. Juice is obtained by hot water washing in a diffuser and discarding fibers, followed by filtration to eliminate solid particle residue from raw agave juices. Filtered juice is thermally hydrolyzed by heating (80 °C) for 8–12 h before refiltering. A second filtration lowers water content. Then, juice is vacuum-evaporated (90 °C) for glycosidic activity denaturation. This results in the end syrup product [23].

Natural aguamiel (juices obtained from fresh or cooked agave “piñas” or cores) can be employed for obtaining high fructose syrup, agave fructans, polysaccharides, biofuel and Maillard compounds [24]. Recent research into the extraction and generation of novel bioactive compounds (for example, saponins and antioxidants) indicates more opportunities for the agave value chain industry [22]. More and more attention has been paid to fructose-rich syrups in recent times, which have become the most demanded sweeteners by the global pharmaceutical and food industries thanks to their technological and functional advantages over sucrose, and their beneficial health effects [11,25] in relation to the bioactive compounds present (fructanes amino and acids) [26]. All of this confers antibacterial properties and antioxidant capacity.

Traditional processing to exploit agave as a source of bioactive compounds and carbohydrate-rich syrups can be performed for direct use or as substrates to yield spirits and hydrolyzed fermented products [21,26].

In recent times, the food industry has paid attention to extract fructopolysaccharides from agave species, which are known as agave fructans (or agavins), because agavins promote human health [27]. ASs can be generated by acid hydrolysis, thermal hydrolysis or glycosidic enzymes from agave fructans [23]. Fructans are the main water-soluble carbohydrate in agave species; they represent > 60% of total soluble carbohydrates [28]. Fructan content in the heads of some agave species falls within the 35–70% dry matter range [29].

The demand for agave fructans in the food industry is increasing because of the technological and prebiotic effects proven by native agave fructans and a considerable degree of polymerization fractions. Agave fructans comprise simple sugars, a complex fructo-oligosaccharides (FOS) and fructans mixture, and linkages β-(2-1) and β-(2-6), including an external (graminans fructans) and internal (neoseries fructans) glucose unit, which varies depending on plant age [30]. Agave fructans are classified as FOS according to the degree of polymerization (DP) (DPs range between 2 and 10) or high DPs (DPs between 2 and 60) [11]. The extraction of agave fructans gives a frequently discarded insoluble dietary fiber-rich by-product [31]. The discarded by-product can be used for developing food ingredients, for example, adding agave ingredients modifies short-chain fatty acid production in granola bars [30].

The use of agave fructans as healthy additives continue to gain interest in the food industry due to their nutritional and technological characteristics, their prebiotic benefits such as soluble dietary fiber, as well as stabilizers and sweeteners, among other applications [32]. The presence of these low molecular carbohydrates makes it possible to obtain prebiotics, fermented products and/or syrups [32].

ASs can be regarded as vegan. As such, manufacturers use them to achieve the same sweet results in their vegan recipes. ASs can also be utilized as a natural sweetener in a wide variety of end products, including prepared beverages, pharmaceuticals, sport drinks, pastry, confection, energy bars, dairy products, sauces, and dressings [33].

The percentage of sucrose replacement with ASs affects both the microstructural rheological and properties of batters, and the physical parameters of baked products. The sensory evaluation of muffins substituted for AS and partially hydrolyzed AS (PHAS) can serve as excellent alternatives for as much as 75% sucrose replacement. Cohesiveness also significantly increases as sucrose substitution levels escalate [34]. Muffin formulations with PHAS present better flavor, color, and texture in particular, and acceptability in general, compared to those formulated with ASs when substituting 100% sucrose [34].

ASs have a different carbohydrate profile with a higher phytochemical potential compared to other sweeteners because more natural compounds are present that display antioxidant activity [35]. A difference in chemical composition is also noted in the same *A. tequilana* syrup samples. This difference lies in the distinct times applied to agave cooking. The color of sweeteners is associated with the content of pigments that possess antioxidant activity. Those with greater antioxidant activity, a higher phenols content and containing pro-anthocyanidins tend to be darker sweeteners [35].

Agave leaves contain non-structural sugars at much lower levels. These levels diminish from the base up to the tip. In *A. tequilana* leaves (fresh weight), the total reducing sugars range lies between 9.4% at the base and 3.3% at the tip. Conventionally, neither plant leaves nor bagasse have been utilized [29], which make them candidates to be employed as fuel. It is possible to use the fibrous waste from agave as several sources, such as straw, paper-making fiber, fertilizers, and baskets [24].

Sustainability issues need to be considered against this backdrop on agave plant versatility. In the food industry, the functional-technical properties of certain food products can be improved by adding bagasse extracts and leaves. By way of example, *A. americana* leaves are employed as powder in steamed yoghurt formulations because a product’s color, texture and viscosity can significantly improve [36]. Calorie and fat content are significantly lower and soluble fiber increases when employing fructans isolated from *A. angustifolia* to replace fat in cookies [37]. Oil- and water-holding capacity are enhanced, which helps to control cookies and can avoid having to add fructans, which means higher yields, meaning larger cookies. Cooky sensory-texture properties also increase, for example, their hardness and color. A sensory analysis shows no differences in the general preference of the formulations that include 10% and 20% fructans as fat substitutes in yoghurts [37].

Bagasse, fibers, and leaves (from stems and leaves) are the principal by-products/residue that the agave industry generates. However, stalks, cuticles and spines are being studied for their high cellulose contents and some bioactive compounds. For the textile industry, although spines and stalks are less commonly employed, they are a relevant source of biocolorants, fibers and bioactive compounds, which can serve as substrates for saccharification. Traditionally, cuticles have been utilized to wrap meat preparations of lots of Mexican dishes and to manufacture paper [38]. Varieties such as *Agave salmiana*, *Agave sisalana* (sisal) and *Agave mapisaga* yield hard fibers, which are highly appreciated because they can be employed to make string and ethnic clothing, and their durability stands out [39].

Bagasse is obtained as fibrous waste after employing stems to produce tequila and mescal, or for agave sap extraction. Bagasse represents approximately 40% of the original stem weight. It comprises both lignin and cellulose. Stems are scrapped to yield fibers and sap (bagasse). They are extracted, considered to be waste and discarded. Some 7,710,520 tons of residual bagasse were produced between 1995 and 2019 [40]. Otherwise, bagasse is an excellent source of bioactive compounds (phenolic compounds, fructans and saponins), sugars, fibers, and other valuable biomolecules [38].

It is noteworthy that the booming agave product market imposes grave environmental consequences. For example, those making mezcal respond to this drink’s meteoric rise from ramping up their wild agave collection. This alarms some environmentalists because they fear that slow-growing populations might not recover. Nevertheless, this issue does not apply to tequila or most commercial agave nectars. This is because only cultivated blue agaves are employed, and growers have to keep up with the increased chemical use on farms [41].

Agave plants’ economic sustainability can extend if expended biomass is converted into useful produce and applied for forage, food, agriculture, ensilage, energy, medicine, environment, cosmetic, aesthetic and textile purposes. The demand for the three principal agave industries (bioethanol, tequila, and fructose syrup) is growing. Moreover, a non-quantified blue agave inventory is expected to result in newly established relationships between agave producers and industry. For example, it is possible to mechanically harvest and employ the whole blue agave plant for biofuel production purposes by employing lignocellulosic materials and sugars without separation [42].

Similarly to plenty of other industrially employed crops, agave is internationally associated with global markets. *Agave tequilana* can deteriorate local agro-ecosystems for being mono-cropped and requiring huge investments being made in agricultural inputs to obtain high yields. It has been estimated that emissions in the order of 700,000 tons of CO_2_eq were emitted in 2014 by the agave tequila chain. Of these greenhouse gas emissions, 44% were directly emitted in agricultural and industrial phases, and the remaining 56% while producing inputs, and transporting and distributing the product. In the agricultural phase, the largest contribution stemmed from using nitrogen fertilizers [43].

It can be argued that the most sustainable agave market options are certified as being free-trade organic products. This guarantees that products are manufactured without pesticides and some occupational standards are followed.

## 4. Quality and Safety Control

*Agave tequilana* Weber var. azul is the blue AS. It is a natural sweet substance obtained through the hydrolysis of the fructans stored in agave plants [44], whose use had led to its wide consumption on the world market, which has also increased the fraudulent use of other syrups. Agave has become a popular sweetener thanks to its low glycemic index and prebiotic effect compared to other honeys and natural syrups [44]. Nevertheless, *Agave tequilana* plants are more popular because they are the sugar source employed to produce tequila. The genus Agave includes more than 210 species, and 159 of the species are ubiquitous in Mexico [40]. Maximum AS production is reached when plants are at least 6 years old, which is the agave plant’s maturity age [44].

The carbohydrate content of ASs is high. They comprise mostly fructose (≥60% total soluble solids), followed by glucose and sucrose traces [11]. Such a carbohydrate composition provides ASs with a low glycemic index. This means that they are sweeter than other syrups with quite high glucose and/or sucrose levels such as sugarcane and maize [4]. Apart from glucose and fructose, some FOS are present in certain ASs in smaller amounts because agavin hydrolysis is incomplete [45]. They are decisive for calculating carbohydrate composition to avoid adulterations from other sugars being added.

Regarding identification, a very useful molecular marker of the adulterant detection, authenticity, origin, and quality of natural sweeteners is carbohydrate fingerprinting. Both the determination of glucose-fructose-sucrose contents and oligosaccharide profiles are methods that establish quality in syrup and honey [46,47,48]. During ASs production, the main agronomic species are the *Agave salmiana* and *Agave tequilana* Weber Blue varieties, with differences in their composition and carbohydrate content [44,49]. The Government of Mexico and agave manufacturers have set Mexican standard rules that do not allow any food additives, ingredients or sugars from other sources that are not agave plants to be used to manufacture commercial ASs [50] and other derivative products, such as tequila and mezcal. The specifications and test methods for products made with the blue AS (*Agave tequilana* Weber var. azul and *Agave Salmiana* spp.) are mentioned in this document, which include the definition of fructans (inulina and FOS), and FOS from 2 to 11 degrees of polymerization, hydrolysis type (chemical, thermal or enzymatic, or their combination). It only accepts a unique degree of quality for AS and it is compulsory to produce AS wholly from agave. The microbiological parameters are the usual ones for this food type: fungi and yeasts (<10 CFU/g), coliforms and *E. coli* (negative), total count bacteria (<100 CFU/g) and *Salmonella* spp. (negative at 25 g).

A different compound in AS is agavins, which are reserve carbohydrates in the agave plant, are formed by fructose polymers and one unique glucose. Nowadays, they are considered to be prebiotic substances and offer several applications (wall material and encapsulating bioactive compounds) [51]. As a result of of their special phytochemical and chemical composition, agavins do not undergo degradation by oral microbiome in either the oral cavity or the small intestine by digestive enzymes. Nonetheless, agavins arrive at the large intestine and are fermented by intestinal microbiota to promote the growth of *Bifidobacterium* sp., *Lactobacillus* sp. and *Saccharomyces Boulardii*, considered the main probiotics [52].

Some authors [52] have explored how agavins affect mice. They have observed that their consumption accelerates body weight loss by microbiota modification and the presence of short-chain fatty acids (SCFA) as determining factors [52]. It is known that agavins favor the host’s health by bringing about certain changes in the activity and/or composition of the intestinal microbiome, considered to be prebiotics [53].

**Figure 2:**
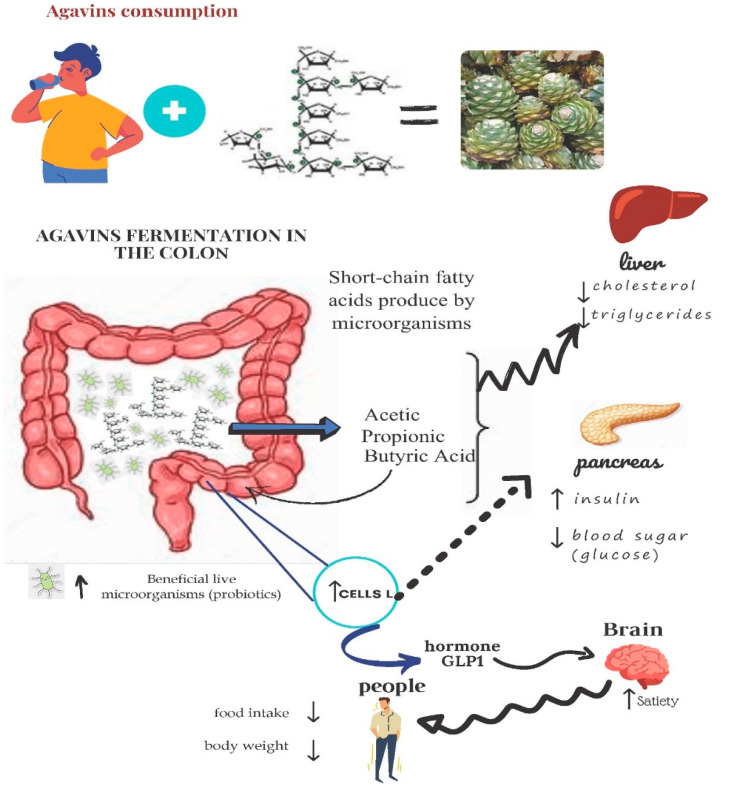
Mechanism by which agavine consumption can generate beneficial effects on health (adapted from [56]).

Their structural complexity lies behind this action on the microbiome. Agavins cannot be degraded by endogenous gastrointestinal enzymes when they pass through the stomach and small intestine. They arrive at the caecum and colon. Here, the saccharolytic microbiotas present at these sites ferment them to produce SCFA, mainly propionate, acetate and butyrate. SCFA are extremely relevant for reducing body weight gain by G-protein-coupled receptors (GPRs). This impacts the secretion of the hormones that are implicated in controlling appetite [54]. Figure 2 shows the mechanism by which agavine consumption can pose beneficial health effects through agave fermentation in the colon. A change in the intestinal microbiome can be brought about by agavins fermentation (SCFA) in the caecum and gut due to a lower pH [15,55].

Gut microbiome growth is not the same for all bacteria [12]. There are three Firmicutes, Proteobacteria and Bacteroidetes phyla, along with five other minor phyla (Tenericutes, Actinobacteria, Cyanobacteria, Defferribacteres, Verrucomicrobia) that dominate the caecal microbiota of mice.

Agavins or oligofructose supplementation is related to distinct communities: with the agavins group, it enhances two genera (*Klebsiella*, *Citrobacter*) and diminishes four (*Ruminococcus*, *Coprococcus*, *Lactobacillus*, *Prevotella*). Oligofructose enhances three (*Faecalibacterium*, *Allobaculum*, *Prevotella*) and diminishes six (Ruminococcus, Enterococcus, *Odoribacter*, *Lactobacillus*, *Desulfovibrio*, *Adlercreutzia*) [52].

Diet supplementation modifies both microbiota activity and caecal microbiota composition. However, the concentration of butyric, acetic, and propionic acids significantly rise by supplementation with oligofructose or agavins, and the pH of caecal content considerably lowers in relation to non-supplemented controls [52]. 

In short, the agave sweetener can be used in lots of food applications as an alternative to sucrose; for instance: muffins [34], cheese [57], cookies [58,59], gummy bear [20,60], ice cream [61], yoghurt [22,62].

## 5. Nutritional Profile and Health Impacts

ASs typically have high soluble solids (>70° Brix) and are primarily made up of fructose and glucose, with small nystose, kestose and sucrose contents [10,63]. The prebiotic action of nystose and kestose in ASs increases its functional value [10,44]. Distinct from other traditional sweeteners, ASs are a source of polyphenols, vitamins and minerals, as shown in Table 2 [64].

In some areas, AS is popular for its low glycemic index (10–27), which is much lower than honey and sucrose [2,16,68], and partially for its carbohydrate pool that contains up to 90% fructose [69]. As AS has a high fructose concentration, it can be used as a sweeter which is better than other many commercially available syrups mostly made up of glucose or sucrose [16]. As a result, not as much AS is required to reach a comparable level of sweetness. This promotes it as a calorie-reduced sweetener. However, such an approach is not without criticism [70].

AS can be controversial if we wish to know if it is a healthier option to sweeteners and table sugar. Syrup proponents argue that it is a better sweetener for diabetics than honey or table sugar thanks to its low glycemic index, and because it creates a smaller blood sugar spike [34,71]. There are, however, additional aspects to bear in mind. According to Jones (2012) [72], the types and content of sugars, macronutrients and ingredients that differ in food products lead to vastly varied glycemic index values. Furthermore, the glycemic index does not correctly reflect food processing and/or cooking methods, individuals’ diet or quantities consumed [1,72]. Nor should the glycemic index be employed as the only criterion to establish a given food or diet’s health effects [1], but ought to be combined with different nutritional factors. Consumers can be misled and end up believing that a low glycemic index allows them to consume more than with conventional sweeteners.

Recent research reports that fructose overconsumption is connected to the liver accumulating fat. This is associated with cardiovascular disease, insulin resistance [73], among other harmful problems [74]. Stanhope et al. (2011) [75] report that those who eat 25% of their daily calories in the form of high-fructose corn syrup (55% fructose, 45% glucose) can present higher triglyceride and cholesterol levels than those who eat pure fructose. However, this is more than what most people eat on a daily basis. It is also noteworthy that fructose is not ingested alone in a typical diet but is often combined with glucose [76].

The way that AS is advertised and how much is consumed may be the most important concerns. There is very little knowledge about the long-term effects of ingesting fructose-high foods or beverages on human health [77]. Given these uncertainties, consumers ought to endeavor to consume energy-dense foods in moderation, including AS. Regardless of the source of sugar, one calorie is one calorie for body fatness alterations [78] The sugars in ASs apparently have the same effect on human weight loss as other sugars do [78]. This means that AS is no more natural than either fruit juice concentrate or high-fructose maize syrup. While enterprises are entitled to sell AS as a sweetener, they ought not to claim that this alternative is more natural or healthier than other widely used sweeteners or sucrose. Making strong claims that favor AS intake should be avoided simply because additional research into fructose and its effects on human nutrition and metabolism is necessary.

## 6. Conclusions

This review explores the potentials of AS as a natural sweetener for human consumption. As consumers show considerable interest in demanding a more natural ingredient in their food, it critically examines the quality characteristics, and nutritional and health impacts of AS. We herein examine the analytical methods that are currently available to characterize sugars in agave to help to confirm its quality and to avoid adulteration. Lucrative industrial agave production raises concerns about ethical considerations that hinder sustainability, especially the environment. Finally, we expect more research to be conducted into AG intake on human metabolism to justify its health claims as a natural alternative to other sugars. In addition, research to improve the industrial process for obtaining AS from agave juice via enzymatic or acid hydrolysis, with the goal of preserving beneficial components (for example, polyphenols, saponins, dietary fiber), while lowering the content of potentially harmful components (for example, fructose), is crucial.

## Figures and Tables

**Table 2:** The typical total phenolic and nutrient composition of traditional common sweeteners (adapted with permission from Edwards et al., 2016, Elsevier) [64] ^1^.

Component	Agave Syrup	Honey	Molasses	Maple Syrup	Carob Syrup	HFCS	Sucrose
Energy (kcal/100 g)	310	304	290	260	248 ^a^	281	387
Water (g/100 g)	23	17	22	32	35 ^a^	24	0
Protein (g/100 g)	0.1	0.3	0.0	0.0	1.4 ^a^	0.0	0.0
Total lipids (g/100 g)	0.5	0.0	0.1	0.1	0.0 ^a^	0.0	0.0
Carbohydrate per difference (g/100 g)	76.4	82.4	74.7	67.0	-	76.0	100.0
Total dietary fibre (g/100 g)	0.2	0.2	0.0	0.0	3.3 ^a^	0.0	0.0
Total sugars (g/100 g)	68.0	82.1	74.7	60.5	63.9 ^a^	75.7	99.8
Minerals (mg/100 g)							
Calcium (Ca)	1	6	205	102	86 ^a^	0	1
Iron (Fe)	0.09	0.42	4.72	0.11	1.10 ^a^	0.03	0.05
Magnesium (Mg)	1	2	242	21	54 ^a^	0	0
Phosphorus (P)	1	4	31	2	239 ^a^	0	0
Potassium (K)	4	52	1464	212	1608 ^a^	0	2
Sodium (Na)	4	4	37	12	113 ^a^	2	1
Zinc (Zn)	0.01	0.22	0.29	1.47	-	0.02	0.01
Vitamins							
Vitamin C (ascorbic acid; mg/100 g)	17	0.5	0	0	-	0	0
Vitamin B_1_ (thiamin; mg/100 g)	0.122	0	0.041	0.066	-	0	0
Vitamin B_2 (_riboflavin; mg/100 g)	0.165	0.038	0.002	1.27	-	0.019	0.019
Vitamin B_3_ (niacin, mg/100 g)	0.689	0.121	0.93	0.081	-	0	0
Vitamin B_6_ (pyridoxine, mg/100 g)	0.234	0.024	0.67	0.002	-	0	0
Folate (µg/100 g)	30	2	0	0	-	0	0
Vitamin A (RAE µg/100 g)	8	0	0	0	-	0	0
Vitamin E ‘α-Tocopherol’ (mg/100 g)	0.98	0	0	0	-	0	0
Vitamin K (phylloquinone, µg/100 g)	22.5	0	0	0	-	0	0
Total polyphenolics (mg GAE/100 mL)	1.292 ^b^	1.935 ^b^	9.195 ^b^	1.494 ^b^	-	0.268 ^b^	-

^1^ Unless otherwise specified, data were taken from the USDA database (2019) [65]. ^a^ Data came from Özcan et al., 2007 [66] and ^b^ St-Pierre et al., 2014 [67]. The enzymatic gravimetric methods 985.29 or 991.43 of the AOAC were applied to determine total dietary fibre content. Abbreviations: HFCS; RAE; retinol activity equivalents, high fructose corn syrup and GAE; gallic acid equivalents.

## Data Availability

Not applicable.
